# Inmunohistochemical detection of pandemic SARS-CoV-2 antigens in lung tissue

**DOI:** 10.7705/biomedica.6132

**Published:** 2022-10-31

**Authors:** Jorge Rivera, Sheryll Corchuelo, Edgar Parra, Eugenio Aladino Meek, Marcela Mercado, Orlando Torres-Fernández

**Affiliations:** 1 Grupo de Morfología Celular, Dirección de Investigación en Salud Pública, Instituto Nacional de Salud, Bogotá, D.C., Colombia Instituto Nacional de Salud Bogotá D.C Colombia; 2 Grupo de Patología, Dirección de Redes en Salud Pública, Instituto Nacional de Salud, Bogotá, D.C., Colombia Instituto Nacional de Salud Bogotá D.C Colombia; 3 Dirección de Investigación en Salud Pública, Instituto Nacional de Salud, Bogotá, D.C., Colombia Instituto Nacional de Salud Bogotá D.C Colombia

**Keywords:** COVID-19, lung, immunohistochemistry, antigens, viral, COVID-19, pulmón, inmunohistoquímica, antígenos virales

## Abstract

The COVID-19 pandemic caused by the SARS-CoV-2 virus has generated globally more than 110.7 million infections and 2.4 million deaths. The severity of this infection can range from asymptomatic, mild to severe.

To know the possible associations between the presence of the virus and histopathological alterations found in tissues of fatal cases of COVID-19, the presence of the virus in the lung tissue of a patient with a clinical history of SARS-CoV-2 infection was evaluated.

Lung tissue was histologically processed for immunohistochemical detection of SARS- CoV-2. In the histopathological study, morphological changes associated with pneumonitis of viral origin were observed. Likewise, the location of the SARS-CoV-2 virus was observed mainly in the cytoplasm of the cells of the inflammatory infiltrate.

## Resumen

La pandemia de COVID-19 causada por el virus SARS-CoV-2 ha generado más de 110,7 millones de infecciones y 2,4 millones de muertes a nivel mundial. Esta infección puede ser asintomática y sus manifestaciones clínicas pueden variar entre leves y graves.

Para conocer las posibles asociaciones entre la presencia del virus y las alteraciones histopatológicas encontradas en los tejidos de casos fatales de COVID-19, se evaluó la presencia del virus en el tejido pulmonar de un paciente con antecedentes clínicos de infección por SARS-CoV-2. La muestra se procesó para la detección inmunohistoquímica del virus.

En el estudio histopatológico, se observaron cambios morfológicos asociados con neumonitis de origen viral. Asimismo, el virus se localizó principalmente en el citoplasma de las células del infiltrado inflamatorio.

In December 2019, a group of patients with pneumonia of unknown origin was identified in Wuhan, Hubei Province, China. The etiological agent responsible for this condition was isolated in lower respiratory tract samples from these patients, identified as a novel coronavirus called SARS-CoV-2, an acronym for severe acute respiratory syndrome coronavirus 2. The World Health Organization (WHO) named this infection coronavirus disease 2019 (COVID-19).

Despite efforts to stop its transmission, the infection spread throughout China, and by January 2020, cases had already been recorded in Thailand, Japan and South Korea. In less than three months, this virus rapidly expanded to, at least, 114 countries, causing more than 4,000 deaths. Finally, by March 11, the WHO declared the COVID-19 outbreak a global pandemic ^1^. By the end of February 2021, 110,7 million infections had been reported, leading to 2,4 million deaths worldwide ^2^.

In Colombia, the first case of COVID-19 was recorded on March 6, 2020, shortly before the WHO declared the pandemic. This case was imported from Italy, the country that at that time had the highest epidemic peak in Europe. Soon after, cases of COVID-19 were diagnosed in travelers from different origins, as well as in many of their close contacts ^3^. By February 2021, there were 2,233,589 cases and 59,118 deaths distributed among the 32 departments of Colombia. A total of 48,6% of infections has been recorded in men, with 63.7% of deaths in this same sex ^4^.

Coronaviruses are positive-sense single-stranded RNA viruses, ranging in diameter from 80 to 220 nm, with a viral envelope that shows 20-nm long projections that resemble a crown.

Coronaviruses have five essential genes, of which four encode structural proteins (N, E, M and S) and one is involved in viral transcription and replication (RNA dependent RNA polymerase, RdRp) ^1^. Within the viral particle, a nucleoprotein (N) wraps the RNA genome to form a tubular structure and the envelope protein (E) surrounds this helical nucleocapsid. Two structural proteins are associated with this viral envelope: the matrix protein (M), which is embedded in the envelope, and the structural protein (S), which is anchored to this structure and is the main target of neutralizing antibodies.

SARS-CoV enters its host cell through the binding S protein to cellular receptors, determining viral tropism and pathogenesis ^5^. It has been suggested that SARS-CoV-2 may share a cellular receptor with SARS-CoV because these two viral strains have similar receptor-binding structural proteins. Different studies have shown that SARS-CoV-2 binds to angiotensinconverting enzyme 2 (ACE2), similar to SARS-CoV ^6^.

Among all currently known RNA viruses, coronaviruses have the largest known genome, capable of causing disease in both animals and humans. Taxonomically, coronaviruses belong to the order Nidovirales, family Coronaviridae and the subfamily Coronavirinae, which is divided into four genera: *alphacoronavirus, betacoronavirus, gammacoronavirus* and *deltacoronavirus*. Currently, six coronaviruses are known to cause disease in humans, including four that are endemic - HCoV-229E, OC43, NL63 and HKU1 - and two that have caused epidemics, SARS-CoV and MERS-CoV. Of these, HCoV-229E and NL63 are part of the genus *alphacoronavirus*, and OC43, HKU1, SARS-CoV and MERS-CoV are part of the genus *betacoronavirus*^1^.

The disease caused by the SARS-CoV-2 has been recorded in patients of different ages, and the severity of the infection can range from asymptomatic to severe. In the latter case, patients present severe acute respiratory syndrome (SARS) or respiratory failure, shock, and multiorgan dysfunction ^7^^,^^8^.

SARS is mainly characterized by pneumonia-like symptoms, being the lung the pathologically most affected organ. However, studies have suggested that SARS is a systemic disease with broad extrapulmonary spread, which results in viral dispersion through respiratory secretions, feces, urine and possibly sweat ^7^^,^^8^.

Immunohistochemical and *in situ* hybridization assays using organs of patients who died from SARS-CoV have shown its presence in the lungs, intestine, liver, distal renal tubules, sweat glands, parathyroid gland, pituitary gland, pancreas, adrenal glands and brain. Through real-time PCR assays, SARS-CoV RNA has been detected in the lungs, intestine, lymphoid nodes, spleen, liver, heart, kidney and skeletal muscle ^7^^,^^9^. However, for SARS- CoV-2, the presence of viral antigens has been described mainly in the upper airways, with abundant immunoreaction in the bronchial epithelium, submucosal glandular epithelium, type I and type II pneumocytes, alveolar macrophages and hyaline membranes in the lung ^10^.

No viral antigens have been observed in the heart, liver, kidney, spleen or intestine. However, in a study that evaluated the tropism profile of SARS- CoV-2 in human cell lines derived from different organs such as lungs (A549, Calu-3, HFL), intestine (Caco2), liver (Huh7), kidney (293T), and muscle (RD), glioblastoma cell lines (U251) and cervical tissue (HeLa), it was found that five of these were susceptible to this infection with significant viral replication. Calu3 and Caco2 cell lines, followed by Huh7 and 293T, showed robust replication while U251 showed modest replication ^11^. This last finding may correlate with the observation that up to 9% of patients with COVID-19 developed confusion or dizziness and some have presented loss of smell and loss of taste ^12^^,^^13^.

The histopathological findings in cases of SARS-CoV-2 are nonspecific alterations. The presence of diffuse alveolar damage in the acute (exudative) and proliferative (fibroproliferative) phases is prominent, including in patients without ventilatory support requirement ^14^^,^^15^. Other alterations in pulmonary morphology have also been observed, including suppurative pneumonia ^14^, hyaline membranes, capillary congestion ^14^, inflammatory infiltrate (acute bronchopneumonia) ^15^, thrombosis ^16^, and chronic inflammation of the trachea, bronchi, and bronchioles ^17^.

**Figure 1.**
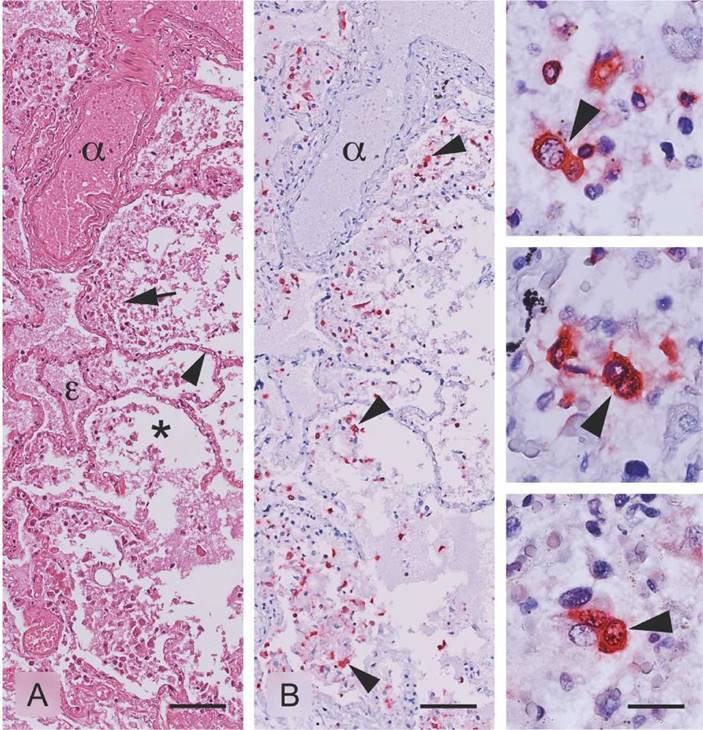
Detection of SARS-CoV-2 antigens in lung tissue of a fatal case with exudative fibrinous pneumonitis and diffuse pulmonary edema. A) Lung tissue in which the alveolus (*), artery (α), and pulmonary edema (£) are observed. The arrow indicates inflammatory cellular infiltrate in the alveolar space. The arrowhead indicates the interalveolar septum where gas exchange takes place, HE stain. B) Arrowheads indicate immunoreaction to SARS-CoV-2 antigens. Images at the right column show viral antigens mainly located in the cytoplasm of inflammatory infiltrate cells, likely macrophages and immune response cells. Immunohistochemistry was performed using the MACH4 Universal AP Polymer Kit™ and visualized with Warp Red. For the detection, a preparation of rabbit anti-SARS nucleocapsid protein antibody (Novus Biologicals-NB100-56576, 1:100 dilution in DaVinci green diluent - Biocare Medical) was used. Scales in A and B = 50 μm; scales in the enlarged right column images = 10 μm

Figure 1 shows lung tissue of a fatal case corresponding to a 61-year- old man who presented sudden respiratory distress and was positive for COVID-19 by real-time RT-PCR using lung tissue samples obtained during the postmortem tissue biopsy. In routine histopathological analysis, acute fibrinous exudative pneumonitis with diffuse alveolar edema suggestive of viral origin was observed. SARS-CoV-2 antigen localization assays via chromogenic immunohistochemistry was observed mainly in inflammatory infiltrate cells and in alveolar areas, confirming SARS-CoV-2 infection of the lungs.
